# Two-Dimensional Perovskite (PEA)_2_PbI_4_ Two-Color Blue-Green Photodetector

**DOI:** 10.3390/nano12152556

**Published:** 2022-07-25

**Authors:** Wei Dou, Ziwei Yin, Yi Zhang, Huiyong Deng, Ning Dai

**Affiliations:** 1 State Key Laboratory of Infrared Physics, Shanghai Institute of Technical Physics, Chinese Academy of Sciences, Shanghai 200083, China; douwei@shanghaitech.edu.cn (W.D.); yinziwei@mail.sitp.ac.cn (Z.Y.); zy_scube@163.com (Y.Z.); 2 University of Chinese Academy of Sciences, Beijing 100049, China; 3 School of Physical Science and Technology, Shanghai Tech University, Shanghai 201210, China; 4 Zhejiang Laboratory, Hangzhou 311100, China; 5 Hangzhou Institute for Advanced Study, University of Chinese Academy of Sciences, Hangzhou 310024, China; 6 Jiangsu Collaborative Innovation Center of Photovoltaic Science and Engineering, Changzhou 213164, China

**Keywords:** 2D nanomaterials, graphene, perovskite

## Abstract

Perovskite materials have been widely used to fabricate solar cells, laser diodes and other photodevices, owing to the advantage of high absorption coefficient, long carrier life and shallow defect energy levels. However, due to easy hydrolysis, it is difficult to fabricate perovskite micro-nano devices. Herein, we developed a water-free device fabrication technology and fabricated a two-dimensional (C_6_H_5_C_2_H_4_NH_3_)_2_PbI_4_ ((PEA)_2_PbI_4_) two-color blue-green light detector, which exhibits high detection performance under the illumination of two-color lasers (λ = 460 nm, 532 nm). Compared with bulk devices, the dark current of the fabricated devices (10^−11^ A) was reduced by 2 orders of magnitude. The peak responsivity and detectivity are about 1 A/W and 10^11^ Jones, respectively. The photodetection performance of the device is basically the same under the two-color lasers. Our results provide a new process to fabricate perovskite microelectronic devices, and the fabricated photodetector shows great application prospects in underwater detection, owing to the blue-green window existing in water.

## 1. Introduction

Regarding photodetection, there exists a blue-green light water window, which is similar to the infrared atmospheric window. The specific performance is that water has the lowest absorption coefficient for the wavelength of 0.45 to 0.55 μm [1]. Blue-green light has the advantages of strong penetrating power that has a more than 90% penetration rate in deep water, low transmission energy consumption, good directionality, and strong anti-interference under water compared to traditional sound waves [2]. Therefore, detectors sensitive and highly responsive to blue-green light are in demand and have promising application prospects [3,4]. Silicon-based photodetectors are usually used for blue-green light detection. However, silicon has a wide spectrum of absorption that needs to be filtered to reduce interference; the absorption coefficient of silicon in the blue band is relatively large, and the penetration depth is small, resulting in low quantum efficiency and responsivity of silicon detectors [5].

As a new generation of optoelectronic materials, perovskites have the advantages of high absorption coefficient, long carrier life, low defect concentration, and shallow defect energy levels [6]. They have been widely applied to LEDs [7], solar cells [8], laser diodes [9], and other fields [10]. There are also many reports on perovskite photoconductive detectors; the MAPbI3 perovskite photoelectric device fabricated by Saidaminov et al. [11] has a ultrafast response speed with a rise/decay time of 30/20 μs; Jiang’s group reported that a detector based on an all-inorganic perovskite α-CsPbI3 has a responsivity of 1294 A/W; [12] Sun’s team successfully manufactured layered organic and inorganic perovskite device with dark currents as low as 1 pA and a detectivity of 10^15^ Jones [13]. However, there are a small number of reports on the practical application of perovskite photodetectors in the visible light band, although experiments have shown that perovskites have extremely high photodetection performance. Preparing large-area high-quality two-dimensional perovskite films is a huge challenge. There are many difficulties in realizing the integrated application of arrayed two-dimensional devices. In particular, materials such as perovskites are easy to dissolve during micro-nano processing.

Herein, we fabricated a two-color blue-green photodetector based on the perovskite (PEA)_2_PbI_4_ (PEPI) due to its high absorption coefficient for this band. We utilized a transfer platform to fabricate the clean and damage-free device, while also ensuring that the entire process is anhydrous. This method solves the problem that lithography cannot be directly used in the preparation of perovskite micro-nano devices. The excellent optoelectronic properties of perovskites enable detectors with high detectivity and responsivity. The peak responsivity of the device is 1.07/0.93 A/W, and the peak detectivity is $2.96\times 10^{11}/2.57\times 10^{11}$ Jones under the irradiation of 460 nm and 532 nm laser, which are superior to conventional silicon-based photodetectors. If we further optimize the preparation scheme of materials and devices, it can be expected that the detection performance of perovskite in the blue-green light band will be greatly improved based on the obtained research results.

## 2. Result and Discussion

Figure S1 shows the schematic diagram of the growth of perovskite crystals using the anti-solvent method [14]. A small container containing 5 mL of a perovskite precursor solution was placed in a large container with 10 mL of volatile anti-solvent dichloromethane. Perovskite crystals would precipitate in the container after the large container was sealed and placed at room temperature for 48 h. The perovskite crystals were washed with a toluene reagent after being taken out and then dried in vacuum.

**Figure 1:**
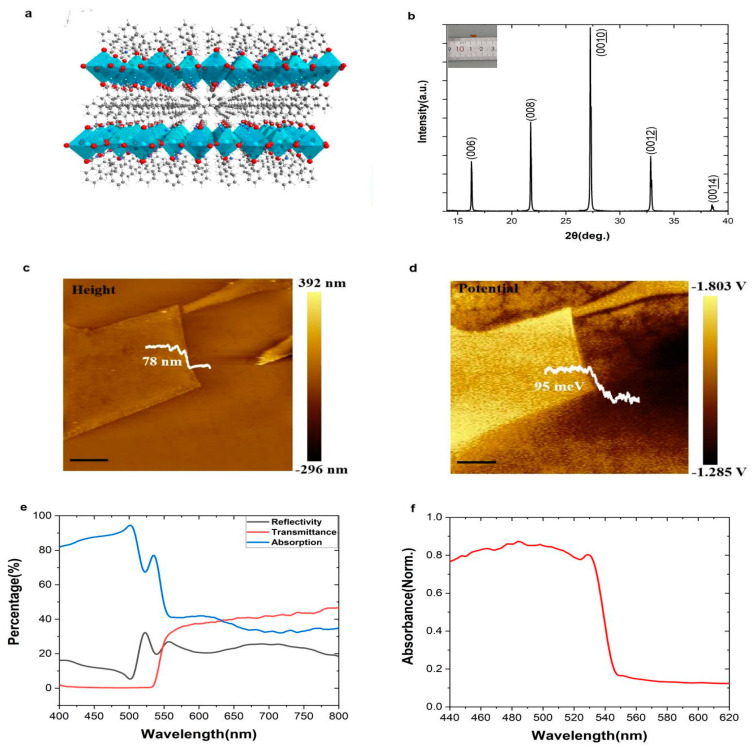
(**a**,**b**) The structural illustration and XRD patterns of 2D(PEA)_2_PbI_4_; (**c**,**d**) AFM and corresponding Kelvin probe force microscopy (KPFM) images of (PEA)_2_PbI_4_ flakes exfoliated on silicon substrate. Scale bar, 5 µm; (**e**,**f**) reflection, transmission and absorption spectra of the (PEA)_2_PbI_4_.

The crystalline model shows that PEPI is a typical layered structure (Figure 1a). The characteristic peaks and peak intensities of PEPI are displayed in the XRD pattern (Figure 1b. The inset figure shows the morphology of the prepared perovskite. A typical size is 5 mm), indicating that the perovskite has good crystallinity and is a pure perovskite phase. We used mechanical exfoliation methods [15] to obtain perovskite flakes of different sizes and heights. The AFM test shows that the size of these perovskite flakes ranges from a few microns to hundreds of microns, and the thickness ranges from tens of nanometers to hundreds of nanometers (Figure 1c and Figure S2). Through the measured transmission spectrum and reflection spectrum, the absorptivity (Figure 1e) of the two-dimensional perovskite in different wavelength bands is calculated without considering the scattering. Further visible light absorption spectrum (Figure 1f) shows that perovskite has a band gap of 2.33 eV, high absorption in the blue-green light band (0.45–0.55 μm), and the absorption cut-off band is about 550 nm, which is a perfect fit for blue and green laser detection.

**Figure 2:**
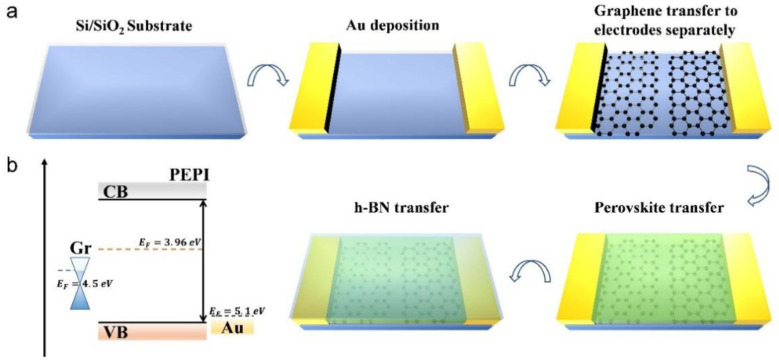
(**a**) A schematic diagram of the fabrication process of the graphene/PEPI/graphene construction; (**b**) interfacial energy alignment diagrams of PEPI, Gr and Au.

Figure 2 shows the fabrication process of the two-dimensional perovskite device. Perovskites are dissolved in the polar solvents such as alcohol and water, which are used in the lithography process [16]. We directly utilize the transfer platform to complete the fabrication of the device. Firstly, a metal mask was used to vapor-deposit gold electrodes. Secondly, two sheets of graphene were transferred to build a channel on the Si/SiO_2_ substrate with a width of about 5 μm and in contact with two electrodes separately. Finally, the PEPI was transferred between the graphene, and then a layer of h-BN was used as a protective layer to isolate water molecules and oxygen [17]. This is a very simple fabrication scheme for micro-nano devices and compared with gold electrodes, graphene has a lower work function [18], which helps to form ohmic contacts between materials and reduces contact resistance [19]. A more general method involves using two sheets of graphene to directly build a channel on the material, or to etch the channel after the perovskite is covered by the graphene. Then, boron nitride is transferred as a protective layer, and finally, gold electrodes are deposited after photolithography. This is suitable for perovskite nanomaterials prepared by various methods, such as nanowires, nanosheets, nanorods, and so on.

**Figure 3:**
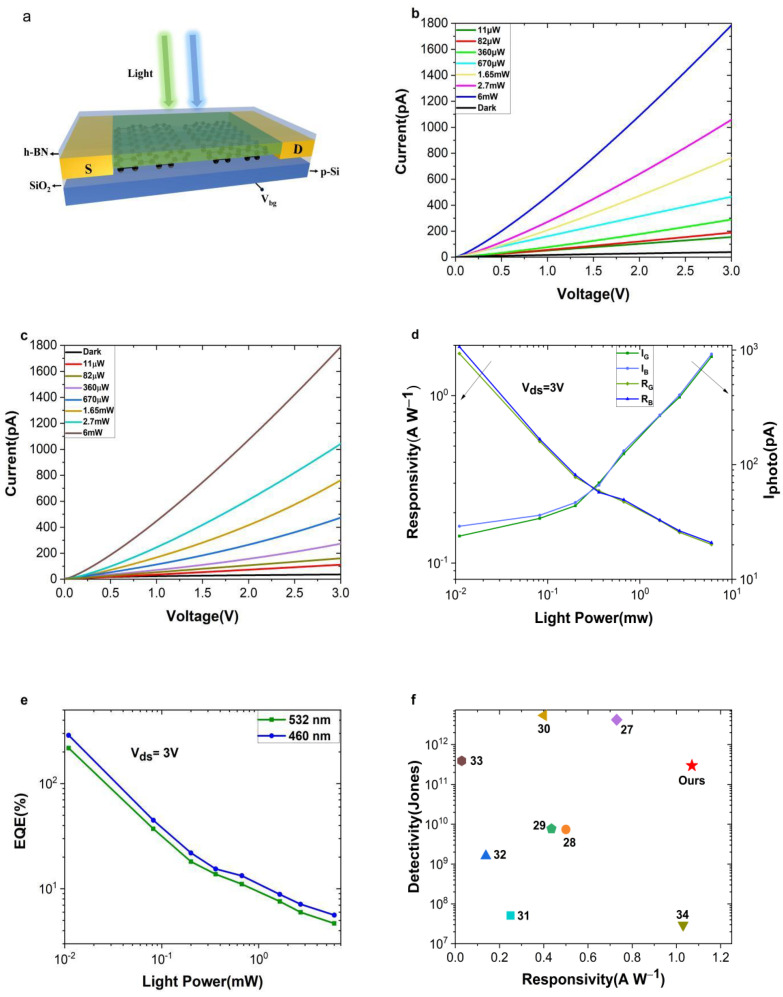
(**a**) A schematic diagram of the Au/graphene/PEPI/graphene/Au photodetector; (**b**,**c**) output characteristics in the dark and under 460 nm (**b**) and 532 nm (**c**) laser illumination at different excitation powers; (**d**) light power-dependent responsivity and photocurrent of the device at bias of 3 V. The subscripts G and B represent 460 nm and 532 nm laser, respectively; (**e**) external quantum efficiency versus light power of the device under 460 nm blue and 532 nm green laser illumination with $V_{ds}=3\;V$; (**f**) comparison of the peak responsivity and detectivity of Gr/PEPI/Gr devices with other reported Si-based photodetectors.

The photodetection performance of the device is given in Figure 3. The model and physical map of the device are shown in (Figure 3a). Figure 3b,c show the *I-V* curve of Gr/PEPI/Gr when photoexcited with a blue (λ = 460 nm) and green laser (λ = 532 nm) under different powers. As the laser intensity increases, the photocurrent increases, and the change trend of the *I-V* curve shows that the graphene and the gold electrode and the perovskite are ohmic contacts. It is noticed that the dark current of the device is as low as $4\times 10^{-11}$ A at a source-drain voltage of 3 V, while the dark current of other 3D perovskite devices is on the order of $10^{-9}\;$A [20,21]. For photoconductive devices, dark current is a very important parameter, which determines the responsivity, detectivity and signal-to-noise ratio of the device. The lower dark current of our device is due to the higher crystallinity and lower defect density of the two-dimensional perovskite crystal [22].

The following formulas [23] are used to calculate the responsivity R and the external quantum efficiency EQE of the device:

(1) $$R\left(\lambda \right)=\frac{I_{on}-I_{off}}{PA_{d}/S}$$

and

(2) $$\mathrm{EQE}\left(\lambda \right)=R\left(\lambda \right)\frac{hc}{q\lambda }$$

where $I_{on}$ and $I_{off}\;$ are photocurrent and dark current, respectively, P is the laser power, $A_{d}$ is the effective photosensitive area, S is the laser spot size, h is the Planck constant, c is the speed of light, q is the charge and λ is the incident laser wavelength. Under the irradiation of 460 nm laser and 532 nm laser (*P* = 0.14 mW/cm^2^), the peak responsivity is 1.07 and 0.93 A/W, and the corresponding external quantum efficiencies are 288% and 217%, respectively. As the laser power increases, the responsivity and external quantum efficiency of the device decrease (Figure 3d,e). The possible reason for this is that the long-lived trap states dominate at low optical powers to provide high photoconductive gain; as higher power impinges on the detector, the effective traps are filled, leading to a decrease of the photoconductive gain [24]. With the synergy of suppressed dark currents and boosted photocurrents in 2D (PEA)_2_PbI_4_, the $D^{*}$ is evaluated by determining the dark noise current [25].

(3) $$D^{*}=\frac{\sqrt{A_{d}f}}{NEP}=\frac{\sqrt{A_{d}f}}{i_{n}/R}$$

where $A_{d}$ is the effective illumination area of the photodetector, f is the electrical band-width, and $i_{n}$ is the noise current.

For photoconductive devices, shot noise is mainly composed of generation-composite noise and photon noise [26], which is approximately given by the following equation:

(4) $$i_{s}=\sqrt{2ei_{d}f}$$

where $i_{d}\;$is the dark current, e is the elementary charge and f is the electrical bandwidth. The size of the shot noise can be approximated by the dark current measured. The 1/f noise is generally considered to be caused by the surface current of the material, of which its noise power spectral density is inversely proportional to the frequency. Under the high frequency limit, 1/f noise is not considered, so the total noise current is approximately calculated by

(5) $$i_{n}=\sqrt{i_{s}{}^{2}+i_{T}^{2}}=\sqrt{2ei_{d}f+\frac{4k_{B}Tf}{V_{ds}/i_{d}}}$$

where $k_{B}$ is the Boltzmann constant, T is the temperature, $V_{ds}$ is the source-drain voltage and $i_{T}$ is the thermal noise current. Due to the low noise and considerable photocurrent, the calculated peak detectivity of the device is $2.96\times 10^{11}$ and $2.57\times 10^{11}\;$Jones under the irradiation of 460 nm blue laser and 532 nm green laser with a 3 V voltage, respectively. Compared with other perovskite photoconductive devices [12,13,27], the detection performance of our photoelectric devices is not so outstanding.

Compared with silicon detection (Figure 3f) shows the peak responsivity and detectivity of related silicon detectors [28,29,30,31,32,33,34,35] in the blue-green band, our device has doubled the responsivity under the premise of approximate detectivity. Under the illumination of a 460 nm and 532 nm laser, the detection performance of the device is almost the same, which shows that dual-channel lasers can be used for simultaneous information transmission to improve the stability and security of signal transmission. There is a certain difference in the responsivity of silicon detectors in different wavelength bands of visible light.

**Figure 4:**
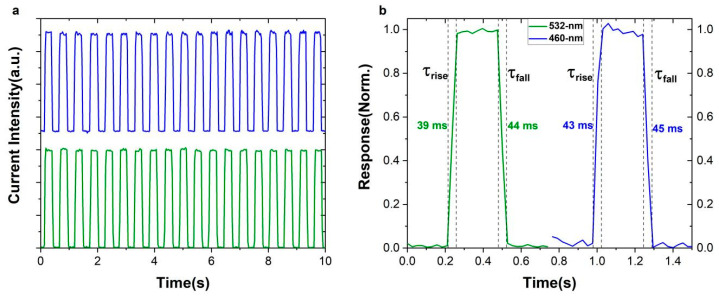
(**a**) The photoswitching characteristics of the Au/graphene/PEPI/graphene/Au device under irradiation of 460 nm and 532 nm laser, and (**b**) zoomed-in switching behavior.

The time-current response curves of the device are tested under the blue and green light, respectively. In a short period of time—about 20 ON/OFF cycles (Figure 4a)—the device shows a certain degree of stability. The rise time (defined from 10% to 90% of the peak photocurrent) and decay time (defined from 90% to 10% of the saturated photocurrent) of the devices are 39/43 ms and 44/45 ms (Figure 4b). However, when we approach 3400 ON/OFF cycles within 1800 s in the extreme test (Figure S4), the performance of the device has significantly decreased. Light, electric field and thermal radiation will inevitably trigger the intrinsic degradation behavior of materials, especially I^−^ and Pb^2+^ in perovskites [36]. On the one hand, I^−^ is easily oxidized to I^0^. I^0^ is not only the carrier recombination center, but, more seriously, it triggers a series of chain chemical reactions, which greatly accelerates the degeneration of the perovskite layer [37]; on the other hand, Pb^2+^ is easily reduced to metallic Pb^0^ when heated or illuminated, which becomes a deep-level defect that seriously affects the photoelectric conversion efficiency and stability of the device [38]; this reveals requirements for improving device life. The perovskite materials with a lower defect density are required, and the redox reaction inside the material needs to be suppressed. Perovskite micro-nano devices also reveal higher requirements in regards to encapsulation technology [39].

## 3. Conclusions

In summary, we have prepared perovskite PEPI nanofilm using the mechanical exfoliation method and an Au/graphene/PEPI/graphene/Au photodetector through dry transfer, combined with graphene. The simple fabrication scheme has been designed to be suitable for low-dimensional perovskite materials prepared through various methods, including nanowires, nanorods, nanoplates and others. Under the irradiation of an 0.14$\;\mathrm{mW}/{\mathrm{cm}}^{2}\;$460 nm and 532 nm laser, the peak responsivity of the device reached 1.07 and 0.93 A/W. The corresponding peak detectivity ranges from $2.96\times 10^{11}$ and $2.57\times 10^{11}$ Jones, respectively. The performance of the device has reached the level of a Gr/Si structure detector, and the responsivity has been doubled, which shows the practical prospects of perovskite-based two-color laser detection in water-related laser communication systems. Based on our findings, further research can be done from two aspects: exploring the combination method and property of the perovskite with other two-dimensional materials, while in order to realize the practical application, it is necessary to prepare the large-area and high-quality two-dimensional perovskite film.

## 4. Methods

Materials.Lead iodide (PbI_2_ > 99.99%), Phenethylamine iodide (PEAI > 99.5%), were purchased from *p*-LED. Dichloromethane (DCM > 99.9%), Butyrolactone (GBL > 99.9%) were purchased from J&K Scientific. All the materials were used as received without further purification.

Synthesis of (PEA)_2_PbI_4**.**_ The PbI_2_ (2.305 g) and PEAI (2.480 g) was mixed in GBL (5 mL) by stirring at 50 °C for 2 h to produce a clear solution. The molar ratio for PbI_2_: PEAI is 1:2. The solution was transferred to a small vial by the syringe with a molecular sieve, and then the small vial was placed into a larger container with methylene chloride (10 mL) as an anti-solvent. The larger container was sealed and allowed to stand at room temperature for 48 h. The dichloromethane volatilized into the perovskite solution, and crystals were precipitated.

Device Characterization. All electrical characterizations of devices were carried out using a Keithley 2602 source meter in ambient conditions. For photocurrent measurement, the devices were illuminated with a fiber-coupled 532 nm and 460 nm laser, equipped with a focusing light system. The illumination power was measured by an optical power meter. The photo-response measurements were conducted with the aid of a light chopper to control the light on/off state.

## Data Availability

The data presented in this study are available on request from the corresponding author.

## Supplementary Materials

The following supporting information can be downloaded at: https://www.mdpi.com/article/10.3390/nano12152556/s1, Figure S1: Preparation of perovskite crystals by anti-solvent method.; Figure S2: Morphology of perovskite of different thickness. Scar bar: 5 µm.; Figure S3: Optical image of the perovskite device.; Figure S4: (A) Transfer characteristics of the device; (B) Long time photoswitching characteristics of the device.
