# Supplementary material for: Two-Dimensional Perovskite (PEA)2PbI4 Two-Color Blue-Green Photodetector
Source: Nanomaterials (Basel). 2022 Jul 25;12(15):2556. doi: 10.3390/nano12152556 (PMC9331230; doi:10.3390/nano12152556)
Supplement: Supplementary file 1 [file nanomaterials-12-02556-s001.zip › nanomaterials-1792762-supplementary.pdf]

nanomaterials

Supplementary information

Two-Dimensional Perovskite $(PEA)_2PbI_4$ Two-Color Blue-Green Photodetector

Wei Dou [1,2,3], Ziwei Yin [1,2], Yi Zhang [1,2], Huiyong Deng [1,2,4,5,*] and Ning Dai [1,2,4,5,6,*]

1 State Key Laboratory of Infrared Physics, Shanghai Institute of Technical Physics, Chinese Academy of Sciences, Shanghai 200083, China; douwei@shanghaitech.edu.cn(W.D.); yinziwei@mail.sitp.ac.cn (Z.Y.); zy_scube@163.com (Y.Z.)
2 University of Chinese Academy of Sciences, Beijing 100049, China
3 School of Physical Science and Technology, Shanghai Tech University, Shanghai 201210, China
4 Zhejiang Laboratory, Hangzhou 311100, China
5 Hangzhou Institute for Advanced Study, University of Chinese Academy of Sciences, Hangzhou 310024, China
6 Jiangsu Collaborative Innovation Center of Photovoltaic Science and Engineering, Changzhou 213164, China
* Correspondence: hydeng@mail.sitp.ac.cn(H.D.); ndai@mail.sitp.ac.cn(N.D.)

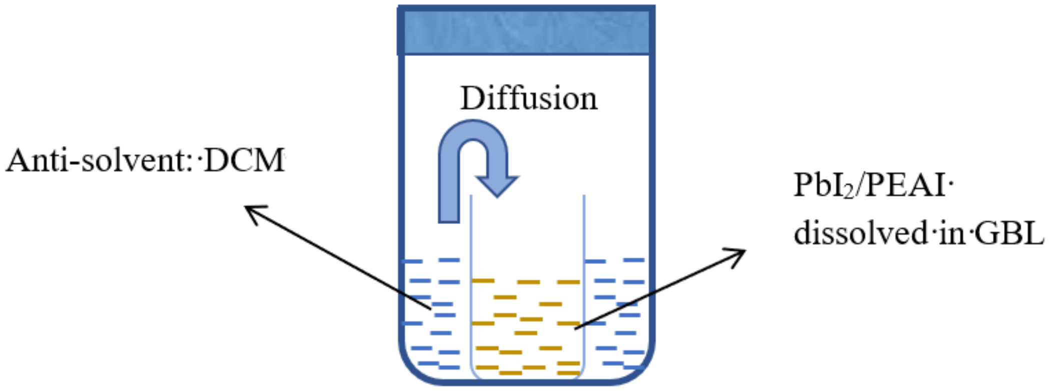

Figure S1. Preparation of perovskite crystals by anti-solvent method.

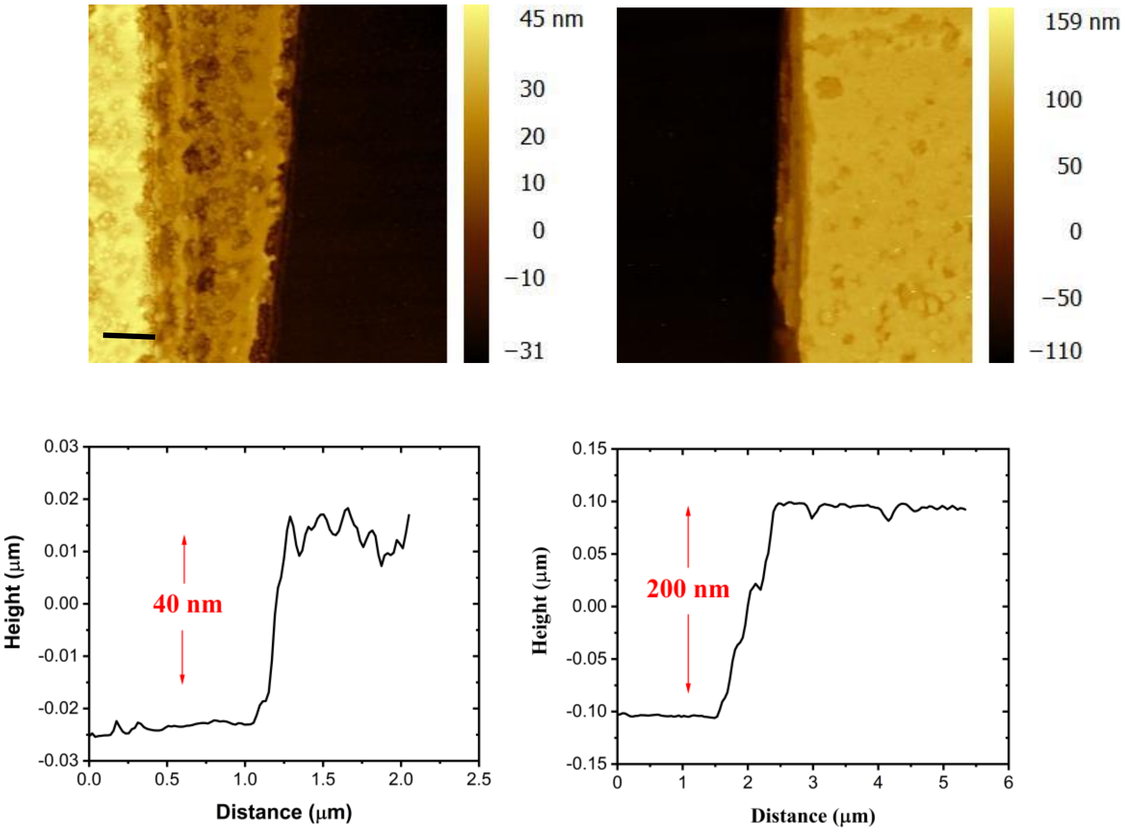

Figure S2. Morphology of perovskite of different thickness. Scar bar: 5 μm.

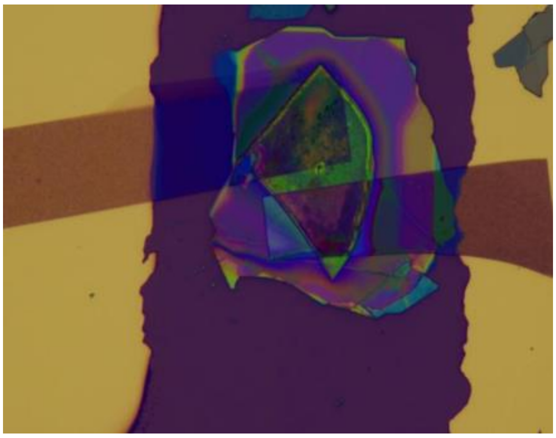

Figure S3. Optical image of the perovskite device. The effective light area of the device is about 103 μm^2. The distance between electrodes is 100 μm.

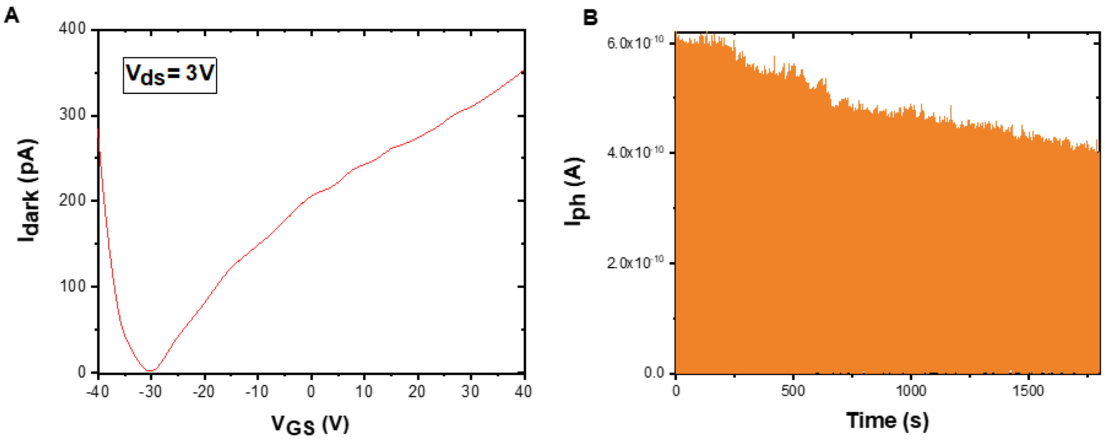

Figure S4. (**A**) Transfer characteristics of the device; (**B**) Long time photoswitching characteristics of the device.
